# Effects of COVID-19 on Pregnant Women and Newborns: A Review

**DOI:** 10.7759/cureus.30555

**Published:** 2022-10-21

**Authors:** Bhavesh M Patel, Deepesh Khanna, Siya Khanna, Vera Hapshy, Pragya Khanna, Payal Kahar, Mayur S Parmar

**Affiliations:** 1 Pediatrics, Gujarat Medical Education & Research Society Medical College, Vadnagar, IND; 2 Foundational Sciences, Nova Southeastern University Dr. Kiran C. Patel College of Osteopathic Medicine, Clearwater, USA; 3 Osteopathic Medicine, Nova Southeastern University Dr. Kiran C. Patel College of Osteopathic Medicine, Fort Lauderdale, USA; 4 Department of Health Sciences, Florida Gulf Coast University, Florida Gulf Coast University, Fort Myers, USA

**Keywords:** covid-19 pregnant, premature labor, covid-positive, covid-19 pandemic, corona virus disease 2019, breast milk coronavirus, intrauterine infection, coronavirus vertical transmission, corona virus, sars-cov-2

## Abstract

Severe acute respiratory syndrome coronavirus 2 (SARS-CoV-2), a novel coronavirus originated in Wuhan, China, and spread all over the world, causing the worst pandemic of the century. The disease has a broad continuum of clinical presentations, from mild to life-threatening. The virus is highly contagious and transmittable to humans. Emerging evidence of its effects on pregnant women and newborns is inconsistent and ever-evolving. Therefore, the objective of this review is to compile the scientific literature on the effects of SARS-CoV-2 coronavirus on pregnancy, pregnant women, and newborns. Data were obtained by several authors using PubMed, MEDLINE, Google Scholar, and Web of Science. "COVID-19", "pregnancy", "vertical transmission", and "newborn" were the search words used to find relevant articles. Most studies suggested pregnant women and newborns are not at additional risk for unfavorable outcomes. Besides, very few studies found newborns who tested positive for SARS-CoV-2 upon delivery from a COVID-positive mother. However, several studies showed no evidence of intrauterine or transplacental transmission of COVID-19 infection. Studies had mixed findings with a few showing the presence of the virus in breastmilk. In conclusion, there is no concrete evidence of additional adverse effects of SARS-CoV-2 on pregnant women and newborns.

## Introduction and background

A series of acute respiratory diseases started in Wuhan, China spreading all over the world at a rapid pace. A novel coronavirus known as severe acute respiratory syndrome coronavirus 2 (SARS-CoV-2) is responsible for the coronavirus disease (COVID-19) [1,2]. SARS-CoV-2 is the third kind of coronavirus causing severe pneumonia after severe acute respiratory syndrome coronavirus (SARS-CoV) and Middle East respiratory syndrome coronavirus (MERS-CoV) [3,4]. On March 11th, 2020, the World Health Organization declared the COVID-19 infection a pandemic [5]. This is a highly contagious and novel coronavirus that has infected approximately 572 million people in the world and as of July 2022, caused 6,390,401 deaths [6]. After the United States, Europe, Western Pacific, and Southeast Asia are the most affected regions [6]. Currently, there are a rising number of cases worldwide. In the United States alone, 89.8 million people have been infected and 1.01 million people have died so far [6]. This virus is directly transmittable between humans via respiratory droplets, contributing to its highly infectious nature. The mortality rate remains the highest in the elderly. Recently, the incidence rate has increased in the young population as well. Currently, the new coronavirus infection has spread throughout the world, and the number of new cases is still on the rise.

The most common presenting symptoms include fever, cough, and shortness of breath [7]. COVID-19 has a range of clinical manifestations from asymptomatic or mild to critical. Most reported cases are those of mild illness; however, data collected regarding the frequency of severe illness is a concern due to detection bias towards these cases since patients who are sick tend to seek a clinical assessment and therefore may be over-represented. Most of the critical cases occurred in the elderly, males, minorities such as African Americans, or populations with underlying medical conditions such as cardiovascular diseases, chronic lung disease, hypertension, and diabetes [8]. There is also evidence that neighborhood-level structural factors such as crowding or larger household membership, as well as psychological stress due to risk factors such as higher unemployment rates, student status, and the presence of chronic illness, may contribute to those disproportionately affected groups [9]. It is thought that existing disparities in access to healthcare and quality of life may also worsen due to the global pandemic [10].

A closer look at vulnerable populations suggests pregnant women may be of concern. However, data regarding the morbidity and mortality of COVID-19-positive pregnant women remains unclear. A child or pregnant woman does not represent an additional risk for adverse outcomes [11-13]. There are several reports on the effect of this novel virus on the respiratory and cardiovascular systems. However, limited research exists on pregnant women and newborns. Pregnant women represent a sub-population having physiological modifications for a prolonged period. A national study conducted by Sutton and colleagues examined 8207 pregnant women in the United States and suggested that those infected with COVID-19 were more likely to suffer from developing severe complications such as ICU admission, mechanical ventilation, and the need for hospitalization. However, this group was not at an increased risk for death compared to their counterparts (non-pregnant women of childbearing age) [14].

Pregnant women are more susceptible to infectious diseases, most notably during the third trimester of pregnancy which is a critical period due to changes in the immune system, and possibly increased changes in lung physiology such as increased respiratory resistance [15]. In addition to presenting with common sequelae of their infectious disease, pregnant women are also at risk of adverse maternal and neonatal complications such as intrauterine growth retardation, premature birth, spontaneous abortion, and vertical transmission of the disease from the mother to the fetus [16]. The pandemics of Spanish flu in 1918, H1N1 in 2009, SARS-CoV, and MERS-CoV showed an increased risk for pregnant women [17,18]. However, no proven cases of vertical transmission (the transmission occurring during the period before or after birth via placental blood, birth canal, or breastfeeding) of SARS-CoV or MERS-CoV have been described [19, 20]. A study conducted by Yan and colleagues on 116 COVID-19-positive pregnant women reported no cases of vertical transmission to the neonates during the third trimester of pregnancy, however, only 65 of these cases were confirmed with laboratory testing [21]. A possible protective mechanism for pregnant women may be due to their placenta and the immunoregulation that occurs during pregnancy [22, 23]. A case has reported elevated IgG and IgM antibody levels to SARS-Cov-2 in neonates born to mothers confirmed with COVID-19, despite negative results for the SARS-CoV-2 RNA real-time reverse transcription-polymerase chain reaction test (RT-PCR) [24]. It is suspected that these findings may be due to postnatal infection arising after contact with COVID-19-infected parents.

In a rapidly changing environment such as this one, it is imperative to further monitor cases and collect data that enables us to better understand the relationship between COVID-19 and pregnant women. The purpose of this review is to investigate the effects of COVID-19 on pregnancy, pregnant women, and newborns. To achieve this, the authors compiled reports on COVID-19-positive pregnant women and the outcomes of the pregnancies.

## Review

Methods

**Figure 1:**
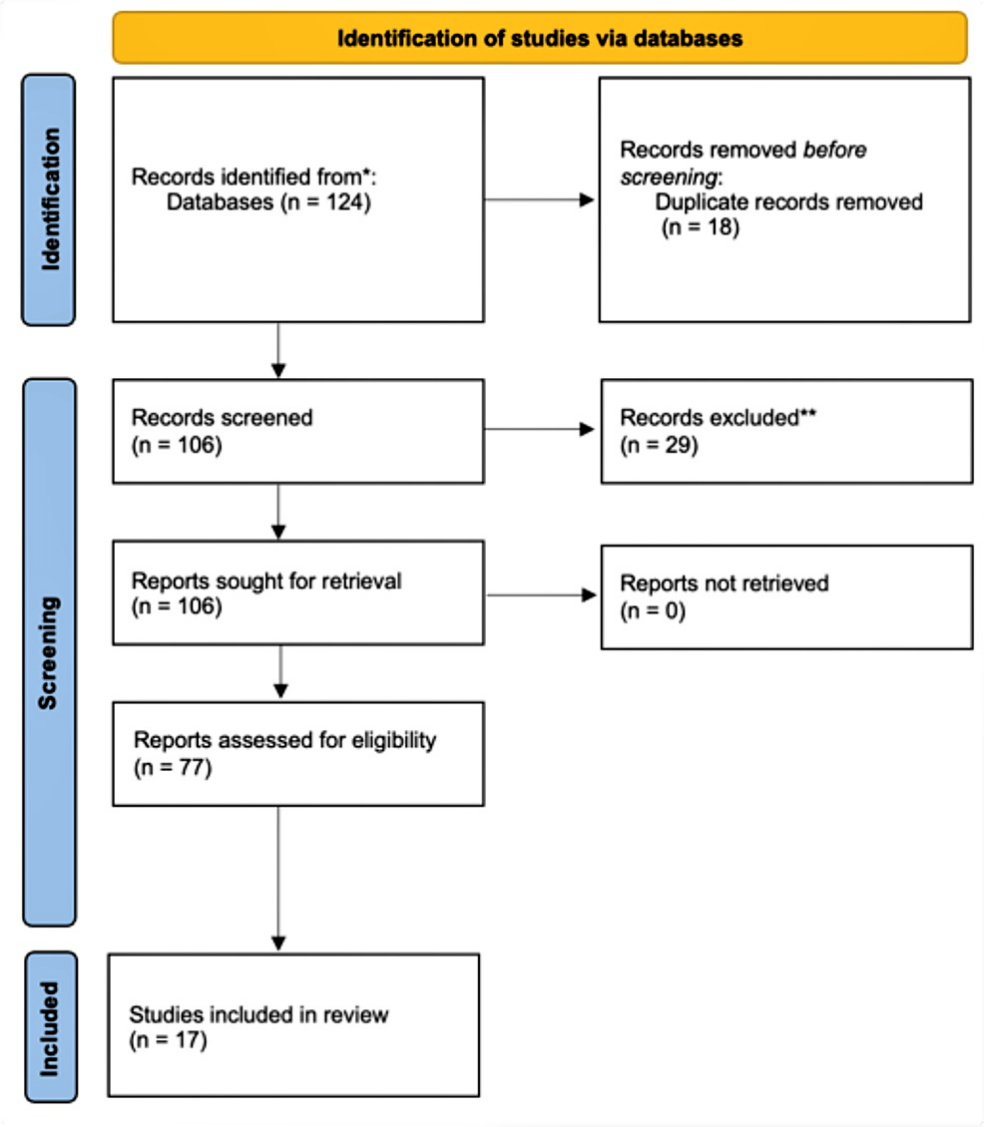
Study selection flowchart *published articles from the PubMed database **articles that do not fit the inclusion criteria

A systematic literature review of the effects of COVID-19 on pregnancy was conducted by using online databases such as PubMed, MEDLINE, Google Scholar, and Web of Science. This review was conducted according to PRISMA guidelines. During the literature search, "COVID-19", "pregnancy", "vertical transmission", and "newborn" were used as search words to find the relevant articles. Publications on the effects of COVID-19 on pregnancy were eligible for inclusion in this systematic review. Statistical data related to COVID-19 cases and deaths were obtained from the Centers for Disease Control and Prevention and the World Health Organization websites. All searches were performed by all the investigators mentioned and re-checked for accuracy on two different occasions. The searches have been restricted to publications on COVID-19 from 2019 to 2022. Most of the eligible studies were excluded if there were any other complications presented other than COVID-19 in the participating pregnant women (Figure 1). 

Results

A total of 17 studies were selected for this review. The studies were divided into five case reports, nine retrospective studies, three cross-sectional studies, and two systematic reviews. The populations analyzed by the researchers in the studies reviewed include pregnant women and neonates in China, USA, Italy, Mexico, India, Iran, Turkey, Spain, and Peru among others. Upon analysis, findings remained variable with regard to the possibility of vertical transmission of COVID-19. Of these studies, 11 out of 19 papers indicated no vertical transmission, while seven studies suggested a possibility of vertical transmission observed via intrauterine transmission, either through the transplacental route or by vaginal delivery. Table 1 displays a summary of the 17 studies analyzed. A majority of the studies provide a low level of evidence as they have a small sample size and do not account for long-term follow-up. Few studies also lacked additional assessments of the virus in cord blood, amniotic fluid, and placental tissue. However, the consensus among the studies suggests that no vertical transmission was observed.

**Table 1:** Summary of studies observing the effects of COVID-19 on pregnant women and newborns

Study	Methodology	Participants	Outcome	Inferences
Lopes de Sousa et al. 2020 [25]	Systematic Review	755 pregnant women and 598 infants	82% of infants were tested for COVID-19, 2% of whom tested positive	No indication of vertical transmission
Ferrazzi et al. 2020 [26]	Retrospective Study	42 COVID-19 positive women who delivered vaginally	2 newborns tested positive for COVID-19 after positive mothers breastfed without a mask due to being diagnosed in the postpartum period; 1 newborn tested positive with vaginal delivery	Vaginal delivery may be associated with intrapartum COVID-19 transmission
Yu et al. 2020 [27]	Retrospective Study	7 COVID-19-positive pregnant women	1 neonate tested positive for SARS-CoV-2 36 hours after birth	No indication of vertical transmission
Alzamora et al. 2020 [28]	Case Report	COVID-19-positive mother and her newborn	Nasopharyngeal swab RT-PCR was positive for SARS-CoV-2 16 hours post-delivery, and serological IgM and IgG were negative for SARS-CoV-2 for mother and neonate	A possible indication of vertical transmission. Pregnant women are considered a high-risk group
Zeng et al. 2020 [29]	Cross-Sectional Study	33 neonates born to COVID-19-positive mothers	Nasopharyngeal and anal swabs were positive for SARS-CoV-2 in 3 neonates. Most seriously ill neonates may be symptomatic due to other causes (sepsis, prematurity, asphyxia) rather than COVID-19 infection	A possible indication of vertical transmission
Nayak et al. 2020 [30]	Retrospective Observational Analytical Study	141 COVID-19-positive pregnant women compared to 836 COVID-19-negative pregnant women	SARS-CoV-2 rRT-PCR test results were positive in 3 out of all neonates initially. Upon retesting 5 days later, the results were negative	No significant effects of COVID infection were observed on maternal and fetal outcomes No indication of vertical transmission
Marin Gabriel et al. 2020 [31]	Retrospective Study	242 COVID-19-positive pregnant women	Nasopharyngeal and oral swab RT-PCR was positive for 11 neonates (9 delivered vaginally), all tested negative in the second sample; 2 neonates that tested negative previously, tested positive in the second sample	No indication of vertical transmission
Moreno et al. 2020 [32]	Retrospective Study	19 COVID-19-positive pregnant women diagnosed during their third trimester and 21 neonates (two sets of twins)	SARS-CoV-2 rRT-PCR test results were negative in all neonates. Prematurity was identified as the most common cause of NICU admission	No indication of vertical transmission
Ayed et al. 2020 [33]	Retrospective Study	185 COVID-positive pregnant women	Nasopharyngeal swab RT-PCR was positive for 2 neonates on day 5	No indication of vertical transmission
Saviron-Cornudella et al. 2020 [34]	Retrospective Study	Of 266 pregnant women, 9% had a previous history of SARS-CoV-2	Newborns of 6 RT-PCR positive pregnant women had negative RT-PCR results	No indication of vertical transmission
Hinojosa-Velasco et al. 2020 [35]	Case Report	COVID-19-positive mother and her newborn	Nasopharyngeal and oropharyngeal swabs were positive for SARS-CoV2 RT-PCR in the mother prior to birth and in the neonate during delivery SARS-CoV2 RNA was detected in the mother’s milk and stool, and infant stool	Intrauterine vertical transmission suspected
Vivanti et al. 2020 [36]	Case Report	COVID-19-positive mother and her newborn	Nasopharyngeal and rectal swabs of the neonate and placenta RT-PCR were positive for both SARS-CoV-2 genes	Transplacental vertical transmission indicated
Mahajan et al. 2021 [37]	Retrospective Study	879 COVID-19-positive pregnant women	Higher risk of pre-eclampsia observed in pregnant women with COVID-19 and multiple gestation pregnancy	Women with multiple gestation pregnancies and COVID-19 require special care
Chi et al. 2020 [38]	Retrospective Study	230 COVID-19-positive pregnant women (154 deliveries)	The most significant neonatal adverse event was premature delivery. The vertical transmission rate calculated by SARS-CoV-2 nucleic acid tests was 3.91%.	Minimal vertical transmission of SARS-CoV-2, however, serum antibodies against SARS-CoV-2 should be tested more frequently
Parsa et al. 2020 [39]	Case Report	COVID-19-positive mother and her newborn	RT-PCR results of the amniotic fluid and neonate (less than 24 hours after birth) were positive for COVID-19	Neonatal positivity for COVID-19 within the first day of life suggests possible vertical transmission
Lu et al. 2020 [5]	Case Report	COVID-19-positive mother and her newborn	SARS-CoV-2 nucleic acid test results from retained amniotic fluid, umbilical cord blood, placenta, and neonatal gastric fluid were negative.	There is no evidence of intrauterine vertical transmission during delivery in the third trimester.
Figueiro-Filho et al. 2020 [40]	Retrospective Study	10,966 cases of COVID-19-positive pregnant patients distributed in 15 countries around the world	The maternal characteristics, clinical symptoms, maternal and neonatal outcomes of almost 11,000 cases of COVID-19, and pregnancy described in 15 different countries are not worse or different from the general population. No vertical transmission was identified in 98.4% of neonates (1,098/1,116).	Pregnant women are not more affected by the respiratory complications of COVID-19 when compared to the outcomes described in the general population. No indication of vertical transmission.

Many researchers reported no indication of vertical transmission. In a study by Ferrazzi et al., two mothers diagnosed during the postpartum period breastfed their newborns without a mask and later tested COVID-19 positive, suggesting the airborne transmission of the virus to the neonate [26]. Moreno et al. sought to study women diagnosed with COVID-19 during their third trimester of pregnancy [32]. They collected a nasopharyngeal swab sample 24 hours after birth in 21 neonates born from COVID-19-positive mothers, and 100% of neonates tested negative. Yu et al. conducted a study and found one neonate tested positive for SARS-CoV-2 36 hours after birth. However, the neonate tested negative two weeks later and was discharged after two negative tests suggesting no vertical transmission occurred [27]. Some researchers reported finding positive SARS-CoV-2 rRT-PCR results in neonates initially. However, they found negative results upon retesting a few days later [30]. Others found SARS-CoV-2 RT-PCR positive pregnant women had newborns with negative RT-PCR [5] or amniotic fluid, cord blood, placental, and neonatal gastric fluid SARS-CoV-2 nucleic acid test results were negative post-delivery [34], both supporting the notion of no vertical transmission of COVID-19 from the mother to the neonate. Lopes de Sousa et al. and Figueiro-Filho et al. conducted large systematic reviews both concluding no vertical transmission was observed in the sample of neonates observed [25, 40].

A few researchers showed the possibility of vertical transmission of the virus [29]. Ferrazzi et al. [26] reported that a COVID-19-positive pregnant woman who vaginally delivered a baby tested positive for COVID-19 despite the mother wearing a surgical mask and the healthcare provider wearing appropriate PPE during labor and delivery. Two studies showed COVID-19-positive newborns, 16 hours and 53 hours after delivery respectively [27, 28]. Both studies reported the presence of COVID-19-positive newborns even after taking all the necessary aseptic precautions by the healthcare professionals and the mothers. A case report in New Mexico discusses a COVID-19-positive pregnant woman who had just given birth to a newborn via cesarian section. Skin-to-skin contact with the mother was restricted for this newborn, and the newborn was fed with synthetic milk until COVID-19 test results were obtained. SARS-CoV2 RNA was detected in the mother’s milk and the stools of both the mother and infant. The newborn tested negative 13 days later while the mother still had positive results, suggesting the possibility of intrauterine vertical transmission [35].

Four case reports suspected the occurrence of vertical transmission between the mother and neonate. Chi et al. [38] calculated a vertical transmission rate of 3.91% confirmed by SARS-CoV-2 nucleic acid tests. On the other hand, Parsa et al. [39] studied the amniotic fluid less than 24 hours after birth and found COVID-19-positive RT-PCR results. Alzamora et al. [28] reported a SARS-CoV-2 positive nasopharyngeal swab in a neonate 16 hours post-delivery. However, IgG and IgM serological levels were negative in both the mother and the neonate likely due to an acute presentation of COVID-19. They explain the low probability of infection during the C-section procedure or postnatally due to the strict isolation measures and sterility procedures implemented immediately post-birth. Vivanti et al. [36] found the nasopharyngeal and rectal swabs of both the neonate and the placenta were positive for both SARS-CoV-2 genes. Signs of acute and chronic intervillous inflammation were also observed in the placenta, consistent with a severe inflammatory status likely due to infection with COVID-19. Maternal and neonatal blood samples were also positive, suggesting transmission occurred via the placenta. Hinojosa-Velasco et al. [35] found nasopharyngeal and oropharyngeal swabs were SARS-CoV2 RT-PCR positive in the mother just before birth, as well as in the neonate after delivery. The newborn was delivered via C-section without premature rupture of the membrane or placenta, along with airborne precautions. Also, SARS-CoV-2 RNA was detected in the mother’s milk, and stool, as well as the infant's stool suggesting vertical transmission occurred. In a retrospective study by Zeng et al. [29], nasopharyngeal and anal swabs were performed on three neonates, and found they were positive for SARS-CoV-2 on the second day of life. They reported the implementation of strict infection prevention protocols during delivery; thus they suspect the likely source of SARS-CoV-2 in the neonates was due to maternal origin. Tables 2-3 further explore pregnancy, maternal, and neonatal outcomes of COVID-19 infection during pregnancy in a few retrospective reviews.

**Table 2:** Maternal outcomes of COVID-19 infection during pregnancy in multiple retrospective reviews

Maternal Complications	Figueiro-Filho et al. 2020 [40]	Chi et al. 2020 [38]	Marín Gabriel et al. 2020 [31]	Nayak et al. 2020 [30]
Cesarian	761/1,119 (68.5%)	124/154 (80.5%)	63/242 (26%)	67/141 (47.5%)
Cesarian for COVID (Severe/Critical)	149/531 (28.1%)	14/154 (9.1%)	6/242 (19.5%)	N/A
Spontaneous Vaginal Delivery	349/1,119 (32.4%)	30/154 (19.5%)	179/242 (73.9%)	66/141 (46.8%)
Operative Delivery	5/241 (2.1%)	N/A	N/A	1/141 (0.7%)
Maternal Hospitalization	558/1595 (34.9%)	N/A	46/242 (19%)	N/A
Maternal Mortality	144/10,987 (1.3%)	1/230 (0.4%)	1/242 (0.4%)	3/141 (2.1%)
Live Birth	847/865 (98%)	156/230 (67.8%)	242/248 (97.6%)	134/141 (95%)
Term	604/772 (78.2%)	130/154 (84.4%)	212/248 (85.5%)	103/141 (73%)
Preterm Birth < 37 weeks	159/764 (20.6%)	24/97 (24.7%)	36/248 (14.5%)	38/141 (27%)

**Table 3:** Neonatal outcomes of COVID-19 infection during pregnancy in multiple retrospective reviews

Newborn complications	Figueiro-Filho et al. 2020 [40]	Chi et al. 2020 [38]	Marín Gabriel et al. 2020 [31]	Nayak et al. 2020 [30]
Low birth weight (<2,500 g)	28/259 (10.8%)	N/A	N/A	39/131 (29.8%)
Respiratory distress syndrome	28/576 (4.86%)	3/55 (5.5%)	26/248 (10.5%)	N/A
Prematurity complications/preterm birth	27/497 (5.43%)	N/A	17/248 (6.9%)	16/134 (11.9%)
Sepsis	1/241 (0.4%)	N/A	N/A	N/A
None	191/241 (79.3%)	N/A	222/248 (89.5%)	
Admission to neonatal intensive care unit	183/992 (18%)	1/42 (2.4%)	28/248 (11.3%)	24/134 (17.9%)
SARS-COV-2-positive	18/1,116 (1.6%)	5/128 (3.9%)	11/230 (4.4%)	3/131 (2.3%)
SARS-COV-2-negative	1,098/1,116 (98.4%)	123/128 (96.1)	219/230 (95.6%)	128/131 (97.7%)
Neonatal death	9/1,137 (0.8%)	2/156 (1.3%)	0/248 (0%)	3/131 (2.3%)

With regards to maternal and fetal outcomes, Lopes de Sousa et al. [25] showed more perinatal complications such as fetal distress, premature rupture of the membranes, and fetal death in COVID-19-positive pregnant women. However, it was difficult to prove that the complications were due to COVID-19.

Discussion

Case Reports/Series

Few case reports explored viral transmission of COVID-19. One study reported the possibility of viral transmission through breast milk [41]. Two other studies reported two newborns tested positive for COVID-19, 16 hours and 53 hours after delivery respectively [27, 28]. Both studies reported that the healthcare and the mothers followed strict guidelines during labor and delivery such as the mother wearing masks the mother, the use of proper personal protective equipment by the medical team, not breastfeeding babies, and separation from the mother. However, the lack of assessment of the presence of viruses in umbilical cord blood or amniotic fluid poses a limitation to the study.

Another study investigated the outcome of asymptomatic COVID-19 infection during the later stage of pregnancy [5]. Pregnant women with asymptomatic COVID-19 infection are rarely reported. However, this study reported the first case of pregnant women with asymptomatic COVID-19 in the Anhui Province of China. The patient had no fever, dyspnea, or cough, and a routine blood examination showed a normal white blood cell count. Before the cesarean section, the patient had received only conventional treatment such as oxygen inhalation, nutritional support, and oral iron therapy. After the cesarean section, she did not breastfeed her infant and received antiviral therapy including interferon α‐2b and Arbidol. Subsequently, the nucleic acid test for the virus turned negative on the third day. Lu et al. [5] assumed that the possible reasons for the successful treatment of the woman were effective drug treatment and young patients with strong immunity. Wang et al. [42] also obtained a positive result by using Arbidol, lopinavir, and ritonavir in treating a pregnant woman with COVID-19 infection. This study showed no evidence of intrauterine or transplacental transmission of COVID-19 infection. This report showed similar results of no intrauterine or transplacental transmission of COVID-19 as other reports published by Chen et al. [17].

According to the preliminary analysis published by Liu et al. [43] on perinatal outcomes of women with coronavirus, pregnancy, and childbirth did not exacerbate the course of action of symptoms of COVID-19 illness. They included 15 of which 11 patients had successful delivery, one vaginal, and 10 cesareans. They reported no cases of neonatal death, neonatal asphyxia, stillbirth, or abortion. The most common symptoms those patients reported were fever (13 out of 15 patients) and cough (nine out of 15 patients). They found the most common abnormal laboratory finding was lymphocytopenia (12 out of 15 patients). Furthermore, the most common finding on chest CT was ground-glass opacity (GGO). However, there was no sign of pneumonia exacerbation after delivery. Based on the above-mentioned study, we can assume that pregnancy and childbirth did not worsen the clinical symptoms of COVID-19 respiratory illness. Another study found similar results on 23 hospitalized pregnant women with confirmed COVID-19 where 15 asymptomatic patients had patchy ground-glass opacity in a single lung lobe and eight symptomatic patients had multiple patchy ground-glass shadows and fibrous stripes. There was a statistically significant difference in lymphocyte percentage. However, the clinical characteristics and CT imaging findings were similar between pregnant women with COVID-19 compared to non-pregnant women with COVID-19 [44]. Another study showed similar results in 10 pregnant women. There were nine singletons and one twin pregnancy. All pregnant women developed mild COVID-19. None of them were severely ill or died. Two patients underwent vaginal delivery, six patients underwent elective cesarean section, and the remaining two patients underwent intrapartum cesarean section. Pulmonary CT imaging showed abnormalities in all the patients after delivery. No neonatal asphyxia was observed. The study concluded that COVID-19 is not an indication for cesarean section. However, pulmonary CT screening would help to reduce the risk of transmission of COVID-19 in the hospital setting during the outbreak period [45].

Another case study reported a newborn who tested negative for the virus and presented with no symptoms but had a high rate of IgM and IgG antibodies against SARS-CoV-2, leukocytosis, and IL-6 cytokines [24]. IgM does not usually cross the placental barrier due to its pentameric structure; it may be possible that IgM was produced in the fetus by way of vertical transmission, however, this remains inconclusive. Another case report discussing neonatal birth to COVID-19-positive mothers described contradictory results with regard to vertical transmission. RT-PCR results of the amniotic fluid and neonate (less than 24 hours after birth) were positive for COVID-19 in one case while the other reported negative PCR results in the amniotic fluid, umbilical cord blood, and neonatal gastric fluid [5, 34]. Together, these two cases exemplify the difficulty in assessing the risk factors associated with vertical transmission of COVID-19 to a neonate. Another study compiled multiple reports on the vertical transmission of COVID-19. The study suggested that most of the reports did not find the virus in the placenta, amniotic fluid, breastmilk, maternal vaginal swab, and a nasopharyngeal swab of the neonate at the time of birth. Three cases in the study reported COVID-19 in neonates. Positive pharyngeal swabs were collected at 36 hours and on Days 2, 4, and 17 of life, and positive tests were based mainly on the presence of IgM and IL-6 in the neonate’s serum. The study concluded that there are no convincing reports that can conclude the vertical transmission of COVID-19 [46].

Cross-Sectional Studies

Most findings report the mortality risk in COVID-19-infected pregnant women as low, as there are only a few cases of patients developing respiratory distress [47]. Pneumonia during pregnancy has also been linked to a greater risk of preterm birth in neonates as compared to the general population [48]. Few studies mentioned in this review are consistent with these findings.

Based on previous studies, infection from seasonal or pandemic influenza A virus (IAV) strain is highly correlated with increased disease severity during pregnancy [49,50]. The experts suggested that the new coronavirus is not an indication of termination of pregnancy. 

Retrospective Reviews

More recently, multiple retrospective studies have been published regarding outcomes of neonates and COVID-19-positive pregnant patients. A study by Lopes de Sousa et al. [25] included 755 pregnant women and 598 infants. More than half of the newborns were delivered by cesarean section (379/755, 65%). Only 493 (82%) infants tested positive for COVID-19 using a reverse transcription-polymerase chain reaction (RT-PCR). They observed fetal distress and gestational diabetes were the most common comorbidities. At the time of admission, the main symptoms and signs presented were fever (53%), cough (42%), and dyspnea (12%). However, 83 pregnant women who tested positive for COVID-19, were asymptomatic, which emphasizes the recommendations given by the Centers for Disease Control and Prevention (CDC) to test high-risk groups who have been in contact with COVID-19-positive individuals [51]. CT scan of the chest was performed in 577 (76%) pregnant women. Out of the 577 women, 538 (93%) showed significant changes in suggestive infection. The laboratory reports showed an increase in C-reactive protein and lymphocytopenia in most of the infected pregnant women. However, the authors suggested, based on the analysis of the reports, that chest x-rays or chest CT scans help in the diagnosis of the disease but do not provide conclusive evidence to confirm the COVID-19 infection. The study also described the effect of infection on childbirth and newborns. A majority of pregnant women delivered their babies by cesarean section. The indication of the cesarean sections were pregnancy-related comorbidities in 148 women, COVID-19 in 103 cases, and not reported in 128 cases. There were eight twins and 590 single births out of a total of 598 newborns. A total of 82% of newborns were assessed for COVID-19, out of whom 2% were positive for the disease. Of the total newborns who tested positive for COVID-19, 20% were premature and 6% were underweight. In most cases, newborns did not suffer from serious complications. However, there were ten neonatal deaths, eight maternal deaths, one spontaneous abortion, one stillbirth, and nine positive COVID-19 cases. In the postpartum period, as the maternal infection was not known, two newborns tested positive for COVID-19 after their mothers breastfed them without wearing masks. The risks present during breastfeeding continue to persist with the potential for neonate exposure to the mother’s respiratory droplets, making it a questionable process. Vertical transmission could still not be ruled out in this group. The authors underscored that the newborns were not checked for the virus soon after birth.

Ferrazzi et al. [26] reported that a COVID-19-positive pregnant woman who vaginally delivered a baby tested positive for COVID-19 despite the mother wearing a surgical mask and the healthcare provider wearing appropriate PPE during labor and delivery.

Chi et. al. [38] described 230 COVID-19-infected patients, 156 of whom delivered vaginally, 10 of who had spontaneous abortions or elective terminations of pregnancy, and 66 of whom had ongoing pregnancies. A total of 34.62% of the pregnant patients had obstetric complications - which included anemia (31.58%), gestational hypertension (13.41%), preeclampsia (12.90%), and gestational diabetes (11.76%). The most common symptomatic presentations were fever (59.05%) and cough (54.76%), followed by postpartum fever (25.51%) and physical discomfort (21.43%). A total of 5.19% of women received mechanical ventilation. Seven women were critically ill. One mother and two newborns died. Vertical transmission to the neonates was reported in 5/128 newborns via a nucleic acid test of SARS-CoV-2 via throat swab. The five infants who tested positive for SARS-CoV-2 were delivered by cesarean section. Otherwise, there were elevated antibody qualitative titers for IgM and IgG against SARS-CoV-2 in eight infants who tested negative via throat swab. Analysis of this data showed that most pregnant patients were mildly ill with fevers and cough the most common presenting symptoms. There was also a significantly larger percentage of cesarean section versus vaginal delivery. The vertical transmission rate calculated by SARS-CoV-2 nucleic acid tests was 3.91% [38]. This review shows that there is limited data to support the presence of vertical transmission, however, the presence of antibodies in infants with negative nucleic acid tests shows that serum antibodies should be drawn more frequently and tested in multiple samples to provide better precision. Although neonatal infection is relatively rare, select mothers elect to abort their child through the worry of infection and premature delivery, and other obstetric complications prompt mothers to undergo cesarean delivery more frequently. The cesarean section seems to benefit both patients and medical staff but there is no clear evidence on whether vaginal delivery or cesarean section is more beneficial [38].

One study investigated the presence of the virus in human breast milk. They examined two SARS-CoV-2 infected mothers. SARS-CoV-2 was extracted from the breastmilk of one of the two mothers on Days 10, 12, and 13. Samples after Day 13 came negative. The authors of the study concluded that milk components might affect RNA isolation as it was determined that the recovery rate of viral RNA in milk moved up with serial dilutions of a SARS-CoV-2 stock [41]. Another study analyzed 64 serial breastmilk samples from women who were infected with SARS-CoV-2 in the United States. Seventeen women had the symptomatic disease. Out of the total 64 samples, one sample tested positive for SARS-CoV-2 by a reverse transcriptase-polymerase chain reaction. The positive sample was collected on the day when a woman became symptomatic. However, one sample collected two days before symptom onset, and two successive samples collected 12 and 41 days later came negative for viral RNA [52]. Furthermore, most of the studies found no evidence of SARS-CoV-2 in human breast milk [53].

Our review largely supports the findings of several studies done to date that have ruled out the vertical transmission of COVID-19. The cases reported in which neonates tested positive for SARS-CoV-2 despite proper sanitation and isolation practices for the mother in the hospital, may be attributed to the asymptomatic presentation of the infected individuals, and possible contraction of the virus in the home environment or hospital after birth. The limitations of many of the studies in this review include a small sample size. Further studies should include larger populations to obtain a more representative set of data. Data collected should encompass pregnant women diagnosed with COVID-19 during all trimesters of pregnancy. Moreover, SARS-CoV-2 RNA testing on amniotic fluid, placental, cord blood, and amniotic fluid in addition to nasopharyngeal swabs should be done to further determine the risk for vertical transmission of COVID-19 to the neonate.

## Conclusions

In summary, based on the current studies, COVID-19 does not have an additional adverse effect on pregnant women, newborns, and the outcomes of pregnancy. There is a good prognosis for pregnant women diagnosed with SARS-CoV-2. The clinical course for pregnant women is like a non-pregnant woman. There is no concrete evidence of vertical transmission of the virus or the presence of the virus in breast milk causing infection in newborns. Our review largely supports the findings of several studies done to date that have ruled out the vertical transmission of COVID-19. The cases reported in which neonates tested positive for SARS-CoV-2 despite proper sanitation and isolation practices for the mother in the hospital, may be attributed to the asymptomatic presentation of the infected individuals, and possible contraction of the virus in the home environment or hospital after birth. However, pregnant women, neonates, and those with gestational complications, or pre-existing comorbidities should be actively monitored in the hospital and receive special care to ensure the prevention of infection. Moreover, SARS-CoV-2 RNA testing on amniotic fluid, placental, cord blood, and amniotic fluid in addition to nasopharyngeal swabs should be done to further determine the risk for vertical transmission of COVID-19 to the neonate. The limitation of many of the studies in this review is that they have a small sample size. Further studies should include larger populations to obtain a more representative set of data. Data collected should encompass pregnant women diagnosed with COVID-19 during all trimesters of pregnancy. Further studies are required to draw any conclusion on the effects of SARS-CoV-2 on pregnant women and newborns and to better understand the nature and course of the virus in these populations. 
